# Comparison of Different Methods of Labor Induction and Their Maternal and Neonatal Outcomes: A Systematic Review and Meta-Analysis

**DOI:** 10.7759/cureus.109016

**Published:** 2026-05-17

**Authors:** Okelue E Okobi, Akinyele Oladimeji, Idayat A Durojaiye, Omowunmi Adewara, Adaora W Mochu, Oluwaferanmi Shofu, Adewale M Adedoyin, Sunday O Arifayan

**Affiliations:** 1 Family Medicine, Larkin Community Hospital Palm Springs Campus, Miami, USA; 2 Family Medicine, IMG Research Academy and Consulting LLC, Homestead, USA; 3 Family Medicine, Alberta Health Services, Edmonton, CAN; 4 Epidemiology and Public Health, University of Alabama at Birmingham, Birmingham, USA; 5 Obstetrics and Gynecology, Newham University Hospital, London, GBR; 6 Family Medicine, University of Perpetual Help System Dalta, Jonelta Foundation School of Medicine, Manila, PHL; 7 Obstetrics and Gynecology, College of Medicine, University of Lagos, Lagos, NGA; 8 Internal Medicine, Lagos State University Teaching Hospital, Ikeja, NGA; 9 Health Sciences, University of Ilorin Teaching Hospital, Ilorin, NGA

**Keywords:** cesarean delivery, dinoprostone, foley catheter, labor induction, misoprostol, tachysystole

## Abstract

Labor induction is a commonly used intervention in obstetric care, yet uncertainty persists regarding the comparative effectiveness and safety of mechanical, pharmacological, and combined methods across different clinical settings. Variability in practice and patient characteristics continues to influence outcomes, and clear comparative evidence remains limited. This systematic review and meta-analysis was conducted in accordance with Preferred Reporting Items for Systematic Reviews and Meta-Analyses (PRISMA) guidelines to evaluate maternal outcomes associated with different labor induction methods. Peer-reviewed studies published between 2010 and 2025 involving adult women with term singleton pregnancies were identified through structured database searches. Quantitative synthesis was performed using R version 4.5 (R Foundation for Statistical Computing, Vienna, Austria, https://www.R-project.org/). Risk ratios were pooled using a random-effects model to compare cesarean delivery, vaginal delivery within 24 hours, and uterine tachysystole across intervention groups.

A total of 25 studies were included in the qualitative synthesis, and four studies with six comparisons were eligible for meta-analysis. The pooled analysis showed no significant difference in cesarean delivery rates between methods, while a higher likelihood of vaginal delivery within 24 hours was observed in selected interventions. A lower occurrence of uterine tachysystole was also identified in specific comparisons. These findings support individualized selection of induction methods based on clinical context and patient characteristics, with emphasis on balancing effectiveness and safety.

## Introduction and background

Labor induction is a common medical intervention used to initiate labor in modern maternity care worldwide [1]. This procedure is performed when continuing the pregnancy poses risks to the mother or fetus, and delivery is considered safer at that time [2,3]. The Royal College of Obstetricians and Gynecologists, World Health Organization, and National Institute for Health and Care Excellence (NICE) guidelines recommend earlier labor induction for various maternal and fetal indications [4]. These guidelines recommend induction when medically indicated, but the outcome must be balanced against the overall benefit of having the baby at that time [5].

There are several clinical indications for labor induction, including post-term pregnancy, pre-labor rupture of membranes (PROM), hypertensive disorders of pregnancy, gestational diabetes, fetal growth restriction, and oligohydramnios [6]. Prolonged pregnancy also increases risks for both the mother and infant [7]. Owing to the risks, clinicians frequently turn to elective labor induction as a management strategy for postdate pregnancies [8].

The process of labor induction consists of two primary components: cervical ripening, a biological change in the structure and composition (collagen), and uterine stimulation [9]. It is important to note that cervical ripening occurs biochemically through the breakdown of collagen, changes in the amount of water in the cervix (the degree of cervical tissue hydration), and mechanical/electrical changes in the extracellular matrix; all of these changes are induced by prostaglandins [10].

Pharmacological induction methods, such as misoprostol (a synthetic prostaglandin E1 analog) and dinoprostone (a naturally occurring prostaglandin E2 analog), promote cervical ripening and labor by enhancing uterine contractility [11]. Mechanical means (i.e., Foley and double-balloon catheters) cause cervical dilation by exerting direct pressure on the cervix and stimulating the release of endogenous prostaglandins [12]. While oxytocin enhances the strength of uterine contractions, its use within the cervix depends upon cervical ripening prior to induction or an unresponsive cervix after initial ripening [13].

The most appropriate method of induction among the various options remains controversial. The various induction methods have been shown to differ in their effectiveness in achieving vaginal delivery, the speed of labor, and their risk profiles (e.g., uterine tachysystole, infection, and maternal discomfort) [14]. Emerging data indicate that mechanical methods are as effective as prostaglandins and, under certain conditions, safer. In contrast, oral misoprostol, particularly when combined with a balloon catheter or prostaglandin, may increase the effectiveness of rapid, successful vaginal delivery [15,16]. However, this potential efficacy must be weighed against the risk of adverse events in the mother or infant [17].

Despite an abundance of randomized clinical trials/controlled studies of the numerically defined outcome variable, clinical practice remains inconsistent because induction methods differ across institutions and regions [18]. Ultimately, induction outcomes can be influenced not only by the method used but, even more importantly, by other patient-related variables such as parity, degree of cervical favorability, body mass index, gestational age, and reason for induction [19,20]. Thus, there is a need for a comprehensive synthesis that marries together efficacy, safety, and patient-specific modifiers. The main objective of this systematic review and meta-analysis is to compare mechanical, pharmacologic, and combined methods of labor induction based on maternal/neonatal outcomes and to evaluate how the clinical scenario/setting affects the optimal induction method.

## Review

Methods

Study Design and Reporting Framework

This systematic review and meta-analysis were conducted in accordance with the Preferred Reporting Items for Systematic Reviews and Meta-Analyses (PRISMA 2020) guidelines to ensure transparency, reproducibility, and methodological rigor [21]. A predefined protocol was developed prior to study initiation to enhance methodological transparency and reduce the risk of reporting bias. However, the review was not prospectively registered in a public database such as PROSPERO.

Search Strategy

A comprehensive literature search of multiple bibliographic databases (e.g., PubMed/MEDLINE, Embase, Cochrane CENTRAL, Scopus, and Web of Science) was performed. The final database search was conducted in December 2025. Studies published between January 2010 and December 2025 were considered, and only studies published in English were included. In addition to the database search, manual screening of the reference lists of included articles was conducted to identify potentially relevant articles. A combination of Medical Subject Headings (MeSH) terms and keywords was used to identify suitable articles for the study. Some examples include labor induction, prostaglandin, Foley catheter, neonatal outcomes, cervical ripening, and maternal outcomes, as shown in Table 1 below. A representative search strategy used in PubMed/MEDLINE was as follows: (“labor induction” OR “induction of labor” OR “cervical ripening”) AND (“misoprostol” OR “dinoprostone” OR “prostaglandin E2” OR “Foley catheter” OR “balloon catheter” OR “oxytocin”) AND (“vaginal delivery” OR “cesarean section” OR “time to delivery” OR “uterine tachysystole” OR “maternal outcomes” OR “neonatal outcomes”). Equivalent search strategies were adapted for other databases using appropriate indexing terms and syntax.

**Table 1:** Overview of search strategy NICU: neonatal intensive care unit

Category	Details
Databases searched	PubMed/MEDLINE, Embase, Cochrane CENTRAL, Scopus, Web of Science
Time frame	2010 – 2025
Language	English only
Study types included	Randomized controlled trials, cohort studies, case-control studies
Search strategy structure	The search strategy was structured using a combination of four key components (#1, #2, #3, and #4)
#1 (population)	Pregnant women, term pregnancy, singleton pregnancy, cephalic presentation
#2 (intervention/exposure)	Labor induction, labor induction, cervical ripening, misoprostol, dinoprostone, prostaglandin E2, Foley catheter, balloon catheter, double-balloon catheter, oxytocin, amniotomy
#3 (comparison)	Mechanical vs pharmacological methods, combination methods, different induction techniques
#4 (outcomes)	Vaginal delivery, cesarean section, time to delivery, uterine tachysystole, postpartum hemorrhage, maternal complications, neonatal outcomes, Apgar score, NICU admission

Eligibility Criteria

The following parameters were considered for inclusion in this review: Randomized controlled trials or observational studies (comprised of cohorts or case-control) that evaluate the comparative effectiveness and safety of different methods of inducing labor and report maternal and/or neonatal outcomes were considered, as long as the population of the study consisted of adult women (≥18 years of age) with singleton, cephalic pregnancies (≥37 weeks gestational age). Studies with special populations (e.g., PROM, previous cesarean delivery, multiple gestations, stillbirth, and/or medically complex pregnant women) were included only if those populations were specifically identified and were analyzed as pre-specified subgroup analyses. All case reports or narrative reviews, editorials, any conference abstracts with insufficient data, animal studies, and studies with the inability to distinguish labor induction from augmentation of labor were excluded from this review.

Study Selection Process

From all records retrieved from the database reference management software, any duplicates were removed. The titles and abstracts of all identified articles were evaluated by two independent reviewers for eligibility, followed by a full-text evaluation of studies that potentially met the eligibility criteria. In the event of any disagreements regarding eligibility decisions, they were resolved through discussion with the other reviewer(s) or a third reviewer. A PRISMA flow diagram was used to document the study selection process.

Data Extraction

A standardized form for data extraction was developed and piloted. The variables extracted from the included studies consist of study-specific information (author, year, country, study design, and study setting); population characteristics (sample size, parity, gestational age, Bishop score, and reason for induction); intervention characteristics (method of induction used, dose used for the induction, and the route used to administer the induction); and outcomes. Maternal outcomes of interest included mode of delivery, time to delivery, uterine tachysystole (more than 5 contractions in 10 minutes), postpartum hemorrhage, and infection, while neonatal outcomes will include Apgar score, admission to the neonatal intensive care unit (NICU), and perinatal morbidity/mortality. Data extraction was performed independently by two different reviewers.

Assessment of Risk of Bias

Methodological quality for included studies was assessed using the Newcastle-Ottawa Scale (NOS) for observational studies. Studies were assigned a risk-of-bias score based on selection, comparability, and outcome assessment [22]. Studies were categorized as low, moderate, or high risk of bias.

Methodological quality for included studies was assessed using NOS for observational studies [22]. For randomized controlled trials, the Cochrane Risk of Bias 2 (RoB 2) tool was used to assess bias across domains, including the randomization process, deviations from intended interventions, missing outcome data, outcome measurement, and selection of reported results [23].

Data Analysis

Quantitative synthesis was conducted using R version 4.5 (R Foundation for Statistical Computing, Vienna, Austria, https://www.R-project.org/) with the meta package. For studies reporting comparable dichotomous outcomes, pooled effect estimates were calculated using risk ratios (RRs) with 95 percent confidence intervals (CIs). A random-effects model with inverse-variance weighting was used to account for between-study variability. Restricted maximum likelihood estimation was used to estimate between-study variance, and the Hartung-Knapp adjustment was applied to improve the reliability of CIs.

Multi-arm studies were included as separate pairwise comparisons. For studies with shared comparator groups, the comparator arm was split evenly across comparisons to avoid double-counting of participants, in accordance with standard meta-analytic practice. Heterogeneity was assessed using the I-squared statistic and the Cochran Q test. Statistical significance was defined as p < 0.05. Forest plots were generated to visually present individual and pooled effect estimates for each outcome.

Results

The comprehensive database search yielded 610 records before duplicates were removed. After removing duplicates (130 records), the remaining 480 records underwent title screening, and 350 records were excluded for reasons such as induction method comparisons, maternal/neonatal outcomes, and failure of augmentation.

Subsequently, 130 full-text articles were screened. Of these, 105 were excluded due to inadequate reporting of outcomes, insufficient differentiation of outcome measures, insufficient data for extraction, or design flaws (e.g., case reports, reviews, abstracts without full data).

**Figure 1:**
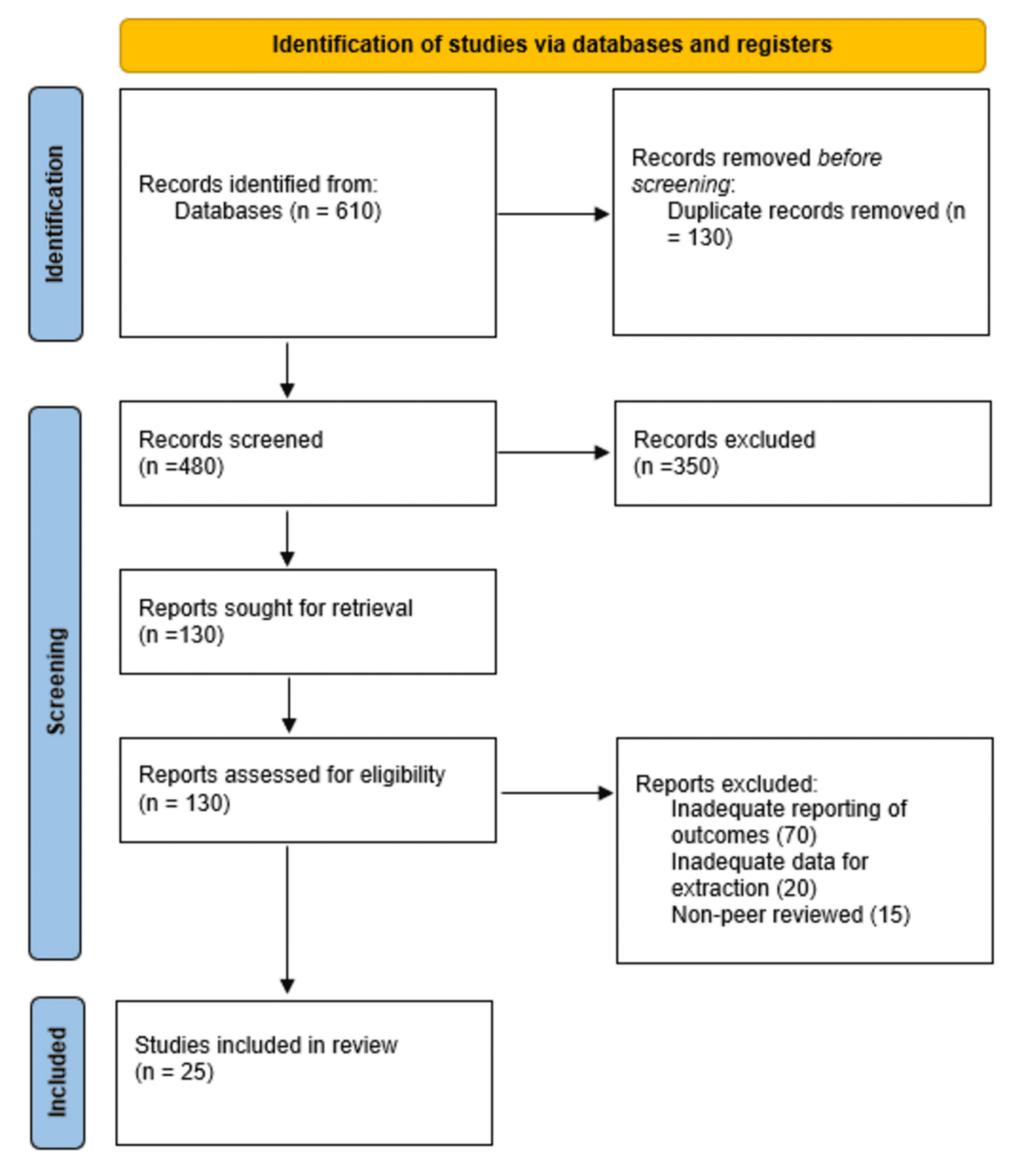
PRISMA flow diagram indicating the study selection and inclusion process This review was conducted in accordance with the Preferred Reporting Items for Systematic Reviews and Meta-Analyses (PRISMA 2020) guidelines [21]. This work is licensed under CC BY 4.0.

A total of 25 studies met the eligibility criteria and were included in the qualitative synthesis. Of these, four studies, contributing six pairwise comparisons, provided sufficient data for inclusion in the quantitative meta-analysis to evaluate the effects of varying labor induction methods on maternal/neonatal outcomes and safety (Figure 1).

Table 2 presents the summary characteristics of the 25 included studies.

**Table 2:** Summary of included studies This table summarizes key characteristics and findings from the studies included in the qualitative synthesis for this review. The information presented, including study design, population, outcome focus, and key findings, was extracted from the original articles and compiled by the authors for clarity and comparability across studies. Review-type articles (e.g., systematic reviews and clinical reviews) were included to provide background context and support the narrative synthesis. These studies were not treated as primary outcome studies and were not included in the quantitative meta-analysis. PROM: pre-labor rupture of membranes, TOLAC: trial of labor after cesarean

Reference	Study design	Population	Outcome focus	Key findings
Laughon et al. (2012) [1]	Retrospective cohort	Contemporary obstetric population	Induction trends and outcomes	Induction associated with varied outcomes depending on clinical indications and patient factors.
Hallén et al. (2023) [2]	Retrospective cohort	Term pregnancies	Outpatient vs inpatient induction	Outpatient misoprostol showed comparable safety and effectiveness to inpatient care.
Frykman et al. (2025) [3]	Retrospective cohort	Women with prior cesarean	TOLAC outcomes	Previous cesarean indication influenced induction success and delivery outcomes.
Kumar et al. (2021) [4]	Prospective cohort	Women undergoing induction	Indications and complications	Induction associated with increased risk of postpartum hemorrhage depending on indication.
Coates et al. (2020) [5]	Systematic review	General obstetric population	Indications for induction	Wide variation in clinical indications contributing to practice variability.
Eminov et al. (2025) [6]	Retrospective cohort	Term pregnancies	Misoprostol vs dinoprostone	Misoprostol effective but associated with higher uterine activity risks.
Zhou et al. (2022) [7]	Retrospective study	High-risk nulliparous women	Cesarean prediction	Identified predictors, including parity and cervical status, influencing outcomes.
Ehikioya et al. (2024) [8]	Randomized Controlled Trial	Pregnant women at 38+0 to 40+6 weeks gestation	Effect of membrane sweeping on need for induction	Single fetal membrane sweeping reduced the need for elective labor induction and increased the likelihood of spontaneous labor without increasing adverse maternal or neonatal outcomes.
Jozwiak et al. (2014) [9]	Cohort study	Prior cesarean pregnancies	Foley catheter induction	Foley catheter considered safe with acceptable vaginal delivery rates.
Berezowsky et al. (2023) [10]	Retrospective cohort	Women with failed induction	Maternal and neonatal outcomes	Failed induction associated with higher cesarean and neonatal complications.
Mancarella et al. (2025) [11]	Retrospective study	Women with failed dinoprostone	Repeat prostaglandin use	Misoprostol improved outcomes after dinoprostone failure.
Manly et al. (2020) [12]	Comparative cohort	Multiparous women	Foley vs prostaglandins	Comparable effectiveness with differing safety profiles.
Germano et al. (2023) [13]	Retrospective cohort	Prior cesarean with unfavorable cervix	Induction outcomes	Increased cesarean risk in unfavorable cervix conditions.
Mlodawski et al. (2021) [14]	Cohort study	Term pregnancies	Misoprostol vs dinoprostone	Misoprostol associated with faster labor but higher uterine tachysystole.
Tuuli et al. (2013) [15]	Retrospective cohort	Induced labor patients	Misoprostol vs Foley	Similar labor progression with differing complication profiles.
Kruit et al. (2020) [16]	Cohort study	PROM pregnancies	Balloon catheter use	Mechanical methods demonstrated favorable safety outcomes.
Hopkins et al. (2021) [17]	Retrospective cohort	Induction patients	Sequential methods	Combination methods improved delivery time but increased complications.
Dorr et al. (2019) [18]	Retrospective cohort	Term pregnancies	Buccal vs vaginal misoprostol	Comparable outcomes with route-based variability.
Li et al. (2023) [19]	Retrospective cohort	Term pregnancies	Cervical length	Cervical length strongly predicted induction success.
Pierce et al. (2018) [20]	Clinical review	General obstetric population	Prostaglandin use	Prostaglandins effective for cervical ripening and induction.
Kashani-Ligumsky et al. (2023) [24]	Cohort study	Multiparous and primiparous women	Misoprostol safety	Misoprostol effective across parity groups with acceptable safety.
Badr et al. (2024) [25]	Retrospective cohort	Suspected macrosomia	Timing of induction	Timing influenced maternal and neonatal outcomes.
Bjorklund et al. (2022) [26]	Retrospective cohort	Obese women	Cesarean risk	Obesity significantly increased cesarean risk after induction.
Huang et al. (2024) [27]	Retrospective study	Term pregnancies	Delivery time prediction	Predictive models improved estimation of induction duration.
Jelks et al. (2023) [28]	Retrospective cohort	Term pregnancies undergoing elective induction	Elective induction at 39 weeks	Elective induction at 39 weeks was associated with improved delivery outcomes without increasing maternal or neonatal adverse events.

Table 3 presents the methodological quality assessment of the included observational studies using NOS [22].

**Table 3:** Quality assessment for observational studies The quality assessment was carried out using the Newcastle-Ottawa Scale (NOS) [22].

Reference	Selection (max 4)	Comparability (max 2)	Outcome (max 3)	Total score (max 9)	Quality rating
Laughon et al. (2012) [1]	4	2	3	9	High
Hallén et al. (2023) [2]	4	2	2	8	High
Frykman et al. (2025) [3]	4	2	2	8	High
Kumar et al. (2021) [4]	3	2	2	7	High
Coates et al. (2020) [5]	3	1	2	6	Moderate
Eminov et al. (2025) [6]	4	2	2	8	High
Zhou et al. (2022) [7]	3	2	2	7	High
Jozwiak et al. (2014) [9]	4	2	2	8	High
Berezowsky et al. (2023) [10]	3	1	2	6	Moderate
Mancarella et al. (2025) [11]	3	1	2	6	Moderate
Manly et al. (2020) [12]	4	2	2	8	High
Germano et al. (2023) [13]	4	2	2	8	High
Mlodawski et al. (2021) [14]	4	2	3	9	High
Tuuli et al. (2013) [15]	4	2	3	9	High
Kruit et al. (2020) [16]	4	2	2	8	High
Hopkins et al. (2021) [17]	4	2	2	8	High
Dorr et al. (2019) [18]	3	2	2	7	High
Li et al. (2023) [19]	4	2	2	8	High
Pierce et al. (2018) [20]	3	1	2	6	Moderate
Kashani-Ligumsky et al. (2023) [23]	4	2	2	8	High
Badr et al. (2024) [24]	4	2	2	8	High
Bjorklund et al. (2022) [25]	4	2	3	9	High
Huang et al. (2024) [26]	3	2	2	7	High
Jelks et al. (2023) [27]	4	2	2	8	High

Table 4 presents the risk-of-bias assessment for the included randomized controlled trial using the RoB 2 tool [23].

**Table 4:** Risk-of-Bias assessment for randomized controlled trial The quality assessment was conducted using the Cochrane Risk of Bias (RoB 2) tool [23].

Reference	Randomization process	Deviations from intended interventions	Missing outcome data	Measurement of outcome	Selection of reported results	Overall risk of bias
Ehikioya et al. (2024) [8]	Low	Low	Low	Low	Low	Low

The randomized controlled trial included in the qualitative synthesis demonstrated an overall low risk of bias across all assessed domains, as assessed using the RoB 2 tool.

Meta Analysis

In this study, four studies comprising six comparative cohorts were included in the quantitative synthesis of labor induction methods. These studies provided extractable dichotomous data for maternal outcomes, including cesarean delivery, vaginal delivery within 24 hours, and uterine tachysystole. Across the included comparisons, a total of 3,166 participants were analyzed, with outcome events recorded for each predefined endpoint. Multi-arm studies contributed more than one comparison and were analyzed as separate pairwise contrasts to retain all relevant intervention groups. Pooled analyses were performed to assess differences in effectiveness and safety across induction methods, using consistent statistical approaches.

Table 5 below presents the characteristics of studies included in the meta-analysis, including study design, intervention comparisons, and extracted outcome data used for quantitative synthesis.

**Table 5:** Characteristics and extracted data of studies included in the meta-analysis Events A and Events B represent the number of participants who experienced the outcome of interest in the intervention group and comparator group, respectively. Total A and Total B represent the total number of participants in each group. RRs were calculated using these values. Only studies that reported comparable interventions and extractable dichotomous outcomes were included in the meta-analysis. Multi-arm studies included more than one comparison and were analyzed as separate pairwise comparisons to preserve all relevant intervention contrasts. Shared comparator groups were handled appropriately to avoid double-counting of participants. VM: vaginal misoprostol, SM: sublingual misoprostol, MVI: misoprostol vaginal insert, DVI: dinoprostone vaginal insert, CR: controlled release, PGE2: prostaglandin E2, RRs: risk ratios

Study	Comparison	Group A (intervention)	Group B (comparator)	Events A	Total A	Events B	Total B	Outcomes included
Eminov et al. (2025) [6]	VM vs dinoprostone	Vaginal misoprostol	Dinoprostone	62	460	171	541	Cesarean
Eminov et al. (2025) [6]	SM vs dinoprostone	Sublingual misoprostol	Dinoprostone	67	455	171	541	Cesarean
Mlodawski et al. (2021) [14]	MVI vs DVI	Misoprostol insert	Dinoprostone insert	149	367	23	114	Cesarean
Manly et al. (2020) [12]	Foley vs PGE2-CR	Foley catheter	Dinoprostone CR	9	95	8	83	Cesarean; vaginal ≤24 h
Manly et al. (2020) [12]	Foley vs PGE2-gel	Foley catheter	Dinoprostone gel	9	95	6	51	Cesarean
Hopkins et al. (2021) [17]	Sequential vs single	Misoprostol + Foley	Misoprostol alone	35	83	73	281	Cesarean

All included studies reported extractable outcome data for at least one predefined outcome. Multi-arm studies were included as separate comparisons to ensure that each intervention contrast was evaluated independently. Studies without comparator groups or without extractable numerical outcome data were excluded from the quantitative synthesis. The included comparisons encompass pharmacological, mechanical, and sequential approaches to induction, enabling evaluation across diverse clinical strategies.

Individual Study Estimates

Table 6 below presents the pooled effect estimate for cesarean delivery across the included comparisons.

**Table 6:** Pooled effect estimates for cesarean delivery RR: risk ratio, CI: confidence interval

Model	RR	95% CI	p-value
Random-effects	0.89	0.44-1.79	0.680

The pooled RR summarizes estimates across heterogeneous intervention-comparator pairs and should not be interpreted as a direct head-to-head comparison between specific induction methods.

**Figure 2:**
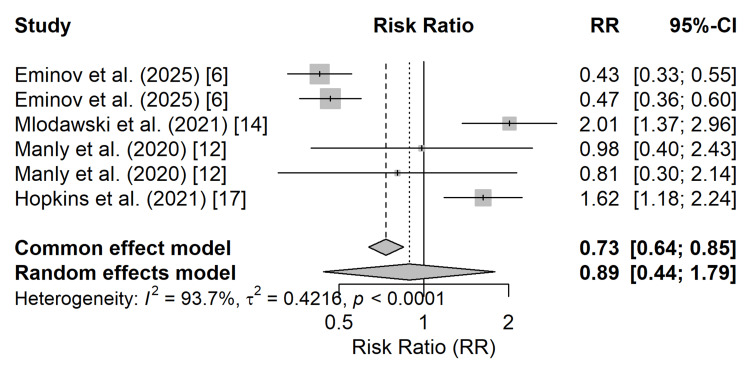
Forest plot of cesarean delivery Each square represents a study-specific estimate, and its size reflects the relative weight in the analysis. Horizontal lines represent 95% CIs, and the diamond represents the pooled estimate. Multi-arm studies appear more than once because each intervention arm was analyzed separately against the same comparator, preserving all relevant comparisons. RR: risk ratio, CI: confidence interval

The findings show that the pooled RR for cesarean delivery was 0.89, with a CI ranging from 0.44 to 1.79. The p-value of 0.680 indicates that the difference was not statistically significant. The wide CI reflects variation across the included comparisons. Figure 2 below illustrates the forest plot for cesarean delivery.

The figure shows that individual estimates are distributed on both sides of the no-effect line. Some comparisons favor the intervention while others favor the comparator. The pooled estimate crosses the reference line, indicating no statistically significant difference in cesarean delivery rates.

Table 7 below presents the pooled effect estimate for vaginal delivery within 24 hours.

**Table 7:** Pooled effect estimates for vaginal delivery within 24 hours RR: risk ratio, CI: confidence interval

Model	RR	95% CI	p-value
Random-effects	1.27	1.10-1.46	0.019

**Figure 3:**
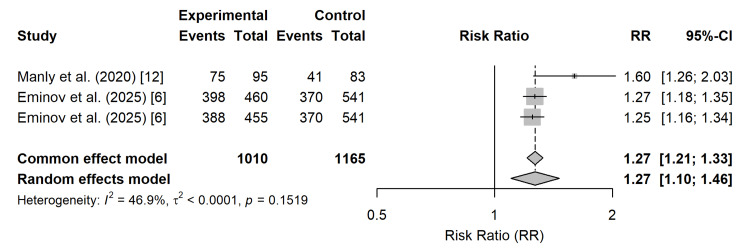
Forest plot of vaginal delivery within 24 hours Multi-arm studies appear more than once because separate intervention arms were analyzed independently against the same comparator. RR: risk ratio, CI: confidence interval

The findings show that the pooled RR for vaginal delivery within 24 hours was 1.27, with a CI of 1.10 to 1.46. The p-value of 0.019 indicates a statistically significant difference. Figure 3 below illustrates the forest plot for vaginal delivery within 24 hours.

The figure shows that all individual estimates lie above the no-effect line. The pooled estimate is also above one, indicating a higher likelihood of vaginal delivery within 24 hours in the intervention group. The estimates follow a consistent direction across comparisons.

Table 8 below presents the pooled effect estimate for uterine tachysystole.

**Table 8:** Pooled effect estimates for uterine tachysystole RR: risk ratio, CI: confidence interval

Model	RR	95% CI	p-value
Random-effects	0.48	0.45-0.51	0.005

The findings show that the pooled RR for uterine tachysystole was 0.48 (95% CI, 0.45-0.51). The p-value of 0.005 indicates a statistically significant reduction. The narrow CI reflects consistency across the included comparisons.

**Figure 4:**
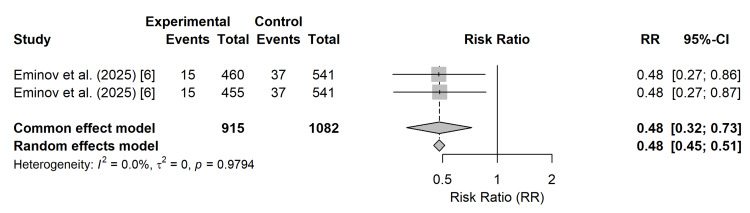
Forest plot of uterine tachysystole Multi-arm comparisons appear more than once because each intervention arm was analyzed separately against the comparator, thereby retaining all relevant data. RR: risk ratio, CI: confidence interval

Figure 4 below illustrates the forest plot for uterine tachysystole.

The figure shows that all estimates are below the no-effect line. The pooled estimate is also below one, indicating a lower occurrence of uterine tachysystole in the intervention group. The close alignment of estimates reflects consistent findings across comparisons.

Study Findings

Efficacy outcomes: The findings of this study indicate that the success rate of labor induction depends on the technique used and the clinical context. The use of pharmacological agents (such as misoprostol) resulted in more women having vaginal deliveries within 24 hours than those using mechanical techniques (such as Foley catheters), indicating that these agents promote cervical ripening more effectively than mechanical methods [6,15,17]. The cesarean section rate did not differ significantly between these groups, suggesting that no single induction technique outperforms the others in all situations [8,12]. The time from the initiation of labor induction to vaginal delivery was shorter for women receiving prostaglandins than for those receiving mechanical methods; however, this apparent advantage may be offset by an increased incidence of uterine hyperactivity [14]. Women who used mechanical methods required more oxytocin for labor augmentation, suggesting that their labor progressed more slowly than women receiving pharmacological means [16]. The failure of labor induction was related to the women’s cervical status and being nulliparous, thus making patient selection a critical component in determining labor induction success [7,19].

Maternal safety outcomes: Maternal safety is an important factor when selecting an induction method. Compared to mechanical methods, pharmacological agents have been shown to have greater uterine activity and hyperstimulation [6,14]. In contrast, mechanical methods (e.g., catheters) have a safer profile than pharmacological agents since they carry no risk of uterine rupture in women who have had previous cesarean deliveries. In women with prolonged labor (greater than 24 hours), the incidence of postpartum hemorrhage was higher than in women with normal labor [9,13]. There is an increased risk of infection complications (e.g., chorioamnionitis) following prolonged or failed labor induction and in cases of pre-labor rupture of membranes [4,10].

Neonatal outcomes: In general, they were similar across all induction methods. However, certain risks tended to vary by the specific intervention. For example, more infants had an Apgar score of less than seven at five minutes, and more infants were admitted to the NICU when there was a failed or prolonged induction, especially when cesarean delivery also occurred [10,25]. The use of pharmacologic induction methods, especially associated with uterine hyperstimulation, has the potential to lead to acidosis in the newborn because of impaired uteroplacental perfusion [6,14]. Nevertheless, most authors did not find a significant increase in serious neonatal morbidity or perinatal death, based on the induction method, when appropriately monitored [8,24]. Therefore, these findings suggest that while efficacy may vary across methods, neonatal safety outcomes would be similar if a clinical protocol is followed [25].

Subgroup analyses have identified additional important differences in neonatal outcome based on maternal characteristics and clinical context. Nulliparous women had consistently higher rates of failed induction and cesarean delivery than multiparous women [7,19]. Likewise, an unfavorable cervix predicted prolonged labor and additional intervention, regardless of the induction method used [15,19]. Obese women were at greater risk for cesarean delivery and longer intervals from induction to delivery due to altered pharmacodynamics and mechanical factors [26]. Additionally, in the case of PROM, mechanical induction methods had a favorable safety profile, including a lower incidence of infection than pharmacologic induction methods [16]. Finally, using oral misoprostol for outpatient induction can yield results comparable to those of inpatient induction, enabling improved resource utilization without compromising safety [2].

Discussion

This meta-analysis shows that cesarean delivery rates did not differ significantly across induction methods, with considerable variability across comparisons. In contrast, the likelihood of vaginal delivery within 24 hours was higher with certain interventions, indicating improved short-term delivery outcomes. A consistent reduction in uterine tachysystole was also observed, with no variability between comparisons. These findings align with the study objective by highlighting differences in effectiveness and safety across induction approaches. However, the analysis was limited to studies with extractable binary outcomes, and important factors such as dosing variations, cervical status, and patient-level characteristics were not consistently captured.

Further, this review suggests that no single method of labor induction can be considered universally superior; rather, different approaches entail trade-offs between safety and efficacy, depending on the clinical context and patient characteristics. Compared with mechanical procedures (i.e., Foley and balloon catheters), the pharmacological agents misoprostol and dinoprostone were more effective in terms of the length of the induction-delivery interval or the rate of labor progress, thereby increasing the chances of vaginal delivery within 24 hours [6,14,17]. However, these pharmacological approaches were associated with increased uterine tachysystole and hyperstimulation [6,14]. According to Berezowsky et al., the use of pharmacologic methods yielded more favorable outcomes in younger women (less than 30 years) and those with lower nulliparity rates. In contrast, reduced response was observed in women with a higher body mass index greater than 25 and advanced maternal age, which was associated with increased rates of intrapartum cesarean section, reported at approximately 60% [10].

In multiparous women, when compared to pharmacologic methods, the use of a Foley catheter is associated with a shorter interval from cervical ripening to delivery. These women are more likely to deliver within 12 to 24 hours and are less likely to require a second method of ripening [12].

Furthermore, the use of dinoprostone gel and oral misoprostol as a second induction cycle was effective after failure of a dinoprostone insert, without a significant increase in adverse effects [11]. Cervical favorability is a critical predictor of successful labor induction outcomes and should be a central consideration in selecting the most appropriate induction method. The Bishop score, which assesses cervical dilation, effacement, consistency, position, and fetal station, remains the most widely used clinical tool for evaluating cervical readiness prior to induction. A higher Bishop score, indicating a favorable cervix, is consistently associated with a greater likelihood of successful vaginal delivery and shorter labor duration, regardless of the induction method employed [19,17].

Women presenting with an unfavorable cervix, characterized by a low Bishop score, typically require cervical ripening interventions to improve their chances of successful induction. Pharmacological agents, such as prostaglandins (e.g., dinoprostone or misoprostol), effectively promote biochemical cervical ripening by inducing collagen degradation and extracellular matrix remodeling, thereby enhancing the cervix’s readiness for labor [10,11].

Mechanical induction techniques yielded comparable vaginal birth rates to the pharmacological agents but slower labor rates compared to the pharmacological agents, and had to use more oxytocin to augment labor [12,16]. Induction methods (i.e., sequential prostaglandin followed by a mechanical method) proved not only faster and more efficient than either method alone but also resulted in a small increase in complications compared with each method used singly [17]. Most neonatal outcomes remain the same regardless of the mode of induction, provided proper monitoring is maintained [24].

Additionally, population-based findings further support variability in induction outcomes across different clinical contexts. Numerous studies have demonstrated that obesity increases the risk for cesarean delivery and lengthens the time between induction and delivery [26]. Additionally, predictive modeling studies have shown that specific characteristics, such as cervical status and type of induction, can be used to estimate time to delivery, enabling personalized and efficient care planning [27]. Elective induction at 39 weeks has been associated with improved delivery outcomes without increasing maternal or neonatal risks, demonstrating that improved timing and standardization of the protocol can enhance the overall effectiveness of the induction process [28].

Mechanistic Interpretation

Variations in cervical and uterine biology can explain differences in outcomes attributable to these three approaches to inducing labor. The effect of misoprostol, an analog of prostaglandin E1, on the cervix is twofold, as it induces biochemical cervical ripening (collagen degradation, increase in water content, and extracellular matrix remodeling) and also induces uterine contractions [10,11]. This action mechanism can explain the effects of misoprostol to hasten labor, and it has likewise been associated with the heightened risk of tachysystole and fetal heart rate anomalies [6,14].

The pathophysiologic rationale for labor induction can be explained by collagen maturity, collagen density, collagen cross-linking organization, metalloproteinase levels, and cervical compliance. More mature, less dense, and well-organized collagen cross-linking was associated with better outcomes. Additionally, increased levels of metalloproteinase and cervical compliance are associated with a more favorable outcome.

Multiparous women tend to have higher induction success rates compared to nulliparous women and a lower tendency to have cesarean sections. Parity and cervical status are the main predictors of successful labor induction [19,20,29]. Nulliparous women had consistently higher rates of failed induction and cesarean delivery than multiparous women [7,19].

Nulliparous women without risk factors have substantially higher rates of complicated birth than multiparous women without a previous cesarean section, even if the latter have multiple risk factors. Grouping women first by parity and previous mode of birth, and then within these groups by the presence of specific risk factors, would provide women with greater, more informed choice, better targeting of interventions, and fewer transfers during labor than grouping by risk factors alone [7,19,30]. However, there is little to no significant difference in the duration of labor induction between nulliparous and multiparous patients [31].

The mechanical induction techniques, by contrast, proceed via direct cervical dilation and trigger the secretion of an endogenous prostaglandin, thereby facilitating a slower process of cervical ripening and reducing the rate of hyperstimulated contractions [12,16]. Oxytocin is mainly a uterotonic agent; it enhances contractile strength. In a cervix that is not ripe, oxytocin causes a contraction. Still, it is not effective in the ripening process of an unripe cervix and must be ripened prior to the commencement of oxytocin [13]. The combination methods of induction exploit both of these (mechanical ripening and pharmacology) to enhance efficiency and delivery time while raising cumulative uterine activity [17].

A critical determinant of induction success is cervical favorability, typically assessed during pelvic examination using the Bishop score. A favorable cervix (higher Bishop score) predicts a higher likelihood of successful vaginal delivery, while an unfavorable cervix (lower Bishop score) warrants cervical ripening prior to induction. Recommendations include prostaglandins (e.g., dinoprostone, misoprostol) or mechanical methods (e.g., transcervical balloon catheter) to improve cervical readiness before stimulating uterine action with oxytocin and/or amniotomy. Ensuring cervical assessment reduces the risk of failed induction and cesarean section [32].

Maternal and fetal monitoring, the well-being of both the woman and the fetus, and labor progress should be reassessed at appropriate intervals based on the induction method and the patient's clinical condition. Once active labor is established, routine maternal and fetal monitoring should be continued [33].

Clinical Decision-Making by Patient Profile

Clinical outcomes varied significantly depending on patient characteristics. The factors that consistently impacted induction time and cesarean delivery rates include nulliparous state (having never delivered a live-born baby) and an unfavorable Bishop score (a score assigned to women that is used to assess their chances of successful vaginal delivery after labor induction) [7,19]. In addition to being nulliparous or having an unfavorable Bishop score, obese women also had lower rates of successful labor induction than their non-obese counterparts. They spent longer in labor than did non-obese women as a result of both altered pharmacokinetics and altered mechanical function associated with obesity [26].

For women who had previously undergone uterine surgery, the mechanical methods of inducing labor were preferred due to the increased risk of uterine rupture if pharmacologic methods of inducing labor were utilized [9,13]. In cases where the membranes were ruptured prior to the onset of labor (i.e., PROM), mechanical methods of inducing labor were associated with a lower risk of infection than prolonged pharmacologic methods [16]. The indication for labor induction (e.g., post-term pregnancy, gestational hypertension, gestational diabetes, suspected fetal compromise, stillbirth, or logistical issues), as well as fetal tolerance, played an important role in choosing which method of inducing labor was used and the overall clinical outcome from labor induction [5,25].

Guideline Alignment

The findings of this study align with WHO and NICE guidance that patient counseling prior to labor induction be individualized based on the patient's overall health, reason for induction, Bishop score, and preferences. Both organizations recommend sweeping the membranes before using pharmacologic or mechanical methods to induce labor [4,5].

Additionally, both organizations support different methods of inducing labor based on the patient's cervical status and maintain that continuous fetal heart rate monitoring is necessary when using pharmacologic methods of inducing labor, even if the patient does not have a history of tachysystole. These findings demonstrate that the methods of inducing labor used in the studies did not differ from those specified by both international organizations [4,5].

Consideration for labor induction is only made when the benefit of inducing labor outweighs that of continuing pregnancy or having an elective cesarean delivery. Common evidence-based indications include post-term pregnancy, PROM, hypertensive disorders, diabetes, suspected fetal compromise, increased risk of adverse fetal outcome, and other maternal conditions affecting pregnancy outcome. Across regions and guidelines, the decision is individualized, incorporating gestational age and fetal status, maternal comorbidities and preferences, and resource availability and setting. This patient-centered framework aligns with best practices by avoiding unnecessary interventions, ensuring timely delivery when indicated, and supporting patient satisfaction in their decision-making capacity [33,34].

Women should be informed why labor induction is being recommended, as well as when, where, and how the procedure will take place. They should also receive information about available support and pain management options. Additionally, choices should be discussed in case the woman decides not to proceed with induction or changes her decision later. The potential risks and benefits of induction in different clinical situations, along with the methods to be used, should be clearly explained. Finally, women should be made aware that induction may not always be successful and understand how this outcome could influence their subsequent care options [34].

Resource and Systems Implications

The options selected for induction are heavily influenced by resource availability. Research has shown that outpatient induction with oral misoprostol provides safety and effectiveness comparable to inpatient methods, while offering lower costs and greater convenience for patients [2]. Nonetheless, when using pharmacological methods, it is important to have access to fetal monitoring and trained personnel available to assist if complications arise (e.g., tachysystole) [13].

Mechanical methods, although slower than pharmacologic methods, may be more appropriate for low-resource settings due to their minimal monitoring requirements and reduced need for medication [9,13]. Staffing levels, drug availability, and institutional policies and procedures have a significant impact on patterns of practice and patient outcomes.

Strengths and Limitations of the Study

The integration of diverse research methods and populations enhances the generalizability of this review. Regardless, there are some limitations. There was considerable variability among patients, induction protocols, and definitions of outcomes of interest. The variability in the cut-off values for Bishop scores (used to assess cervical favorability for labor induction), medication dosing schedules, and the range of indications for inducing labor (e.g., PROM, post-term pregnancy, and high-risk conditions) makes it difficult to draw direct comparisons among studies. Furthermore, the potential bias and confounding associated with an observational design remain an issue, even though many of the studies were deemed high-quality methodologically.

Implications for Future Research

Future research should include stratified analyses by parity, cervical status, and specific clinical indications. Additionally, standardizing outcome measures is an important step toward developing consensus on how to measure uterine tachysystole and neonatal morbidity. There is also a need for additional research in the high-risk populations of obese women and women with a prior cesarean birth. Comparing and evaluating the use of outpatient induction pathways and patient-centered outcomes (pain, satisfaction, experience, etc.) should also provide additional information for making clinical decisions regarding care.

## Conclusions

This study highlights that different methods of labor induction are associated with varying patterns of effectiveness and safety. Pharmacological approaches were linked with a higher likelihood of achieving vaginal delivery within a short time frame. In contrast, mechanical methods showed comparable delivery outcomes with a more favorable safety profile in selected settings. The findings also indicate that cesarean delivery rates did not differ meaningfully across methods, suggesting that no single approach is consistently superior. These results support the need for individualized clinical decision-making based on patient characteristics and clinical context. Future research should focus on standardized outcome reporting and incorporate key variables such as cervical status, parity, and dosing protocols to improve comparability and guide clinical practice.
